# *Bifidobacterium adolescentis* as a key member of the human gut microbiota in the production of GABA

**DOI:** 10.1038/s41598-020-70986-z

**Published:** 2020-08-24

**Authors:** Sabrina Duranti, Lorena Ruiz, Gabriele Andrea Lugli, Héctor Tames, Christian Milani, Leonardo Mancabelli, Walter Mancino, Giulia Longhi, Luca Carnevali, Andrea Sgoifo, Abelardo Margolles, Marco Ventura, Patricia Ruas-Madiedo, Francesca Turroni

**Affiliations:** 1 Laboratory of Probiogenomics, Department of Chemistry, Life Sciences, and Environmental Sustainability, University of Parma, Parco Area delle Scienze 11a, 43124 Parma, Italy; 2 Department of Microbiology and Biochemistry of Dairy Products, Instituto de Productos Lácteos de Asturias – Consejo Superior de Investigaciones Científicas (IPLA-CSIC), Villaviciosa, Asturias Spain; 3 Instituto de Investigación Sanitaria del Principado de Asturias (ISPA), Oviedo, Asturias Spain; 4 Microbiome Research Hub, University of Parma, Parma, Italy; 5 GenProbio Srl, Parma, Italy; 6 Stress Physiology Laboratory, Department of Chemistry, Life Sciences and Environmental Sustainability, University of Parma, Parma, Italy

**Keywords:** Microbiology, Microbial genetics

## Abstract

Gamma aminobutyric acid (GABA) is the principal inhibitory neurotransmitter playing a key role in anxiety and depression disorders in mammals. Recent studies revealed that members of the gut microbiota are able to produce GABA modulating the gut–brain axis response. Among members of the human gut microbiota, bifidobacteria are well known to establish many metabolic and physiologic interactions with the host. In this study, we performed genome analyses of more than 1,000 bifidobacterial strains publicly available revealing that *Bifidobacterium adolescentis* taxon might represent a model GABA producer in human gastrointestinal tract. Moreover, the in silico screening of human/animal metagenomic datasets showed an intriguing association/correlation between *B. adolescentis* load and mental disorders such as depression and anxiety. Interestingly, in vitro screening of 82 *B. adolescentis* strains allowed identifying two high GABA producers, i.e. *B. adolescentis* PRL2019 and *B. adolescentis* HD17T2H, which were employed in an in vivo trial in rats. Feeding Groningen rats with a supplementation of *B. adolescentis* strains, confirmed the ability of these microorganisms to stimulate the in vivo production of GABA highlighting their potential implication in gut–brain axis interactions.

## Introduction

Gamma-Aminobutyric acid (GABA) is a non-protein amino acid that is widely distributed in plants, animals and microorganisms^1,2^. GABA is synthetized by a pyridocal-5′-phosphate (PLP)—dependent glutamate decarboxylase (GAD) enzyme by irreversible α-decarboxylation of l-glutamate and consummation of one cytoplasmic proton^1,2^. GABA has several well-known physiological and psychological functions. Different studies highlighted that it is predominantly present in the brain where it acts as a major inhibitory neurotransmitter in the mammalian central nervous system (CNS)^1,2^. Specifically, dysfunctions in GABA metabolism are involved in anxiety and depression^3–5^. Furthermore, it is involved in the regulation of blood pressure and heart rate and plays a role in the perception of pain and anxiety^5,6^. Other potential health benefits of GABA are control of growth hormone secretion, protection against glycerol-induced acute renal failure in rats and anti-proliferative activity^7^.

Recently, the term “psychobiotic” has been introduced to designate live bacterial strains, including lactobacilli and bifidobacteria, which are able to influence the CNS function^8^. There are several compounds produced by these bacteria, such as proteins, peptides and components of cell wall that are potential mediators between bacteria and their hosts. Neurotransmitters, such as GABA, represent an example of neuroactive molecules produced by psychobiotics and members of the human gut microbiota that have been found to modulate neural signals which affect neurological and psychiatric parameters, as well as sleep, appetite, mood and cognition^8^. Genetically, it has been found the presence of *gad* genes, predicted to encode for glutamate decarboxylase or glutamic acid decarboxylase, in the genomes of Lactic Acid Bacteria (LAB) and bifidobacteria that are supposed to be responsible of the GABA production^5,9–12^. Recent studies revealed that the increased level of GABA in the human gut could be derived by the ability of the intestinal microbiota or ingested probiotic, such as bifidobacteria and lactobacilli, to metabolize dietary monosodium glutamate (MSG)^5,9–12^. Nevertheless, the ability to produce GABA by gut-derived bifidobacteria strains remains poorly studied. Until now, only three bifidobacterial species, such as *Bifidobacterium dentium*, *Bifidobacterium longum* subsp. *infantis* and *Bifidobacterium adolescentis* were shown to produce GABA by means of in vitro studies^5^.

The aim of this study is to understand if the production of GABA in bifidobacteria is a strain-specific feature, analyzing the genomic sequence of 1,022 bifidobacterial strains belonging to the currently known 77 *Bifidobacterium* taxa, representing 70 species and seven subspecies, coupling the in silico information with an in vitro measurements of GABA levels generated by these bacteria. Notably, the production of GABA by those *B. adolescentis* strains displaying the highest in vitro GABA-synthesis performance was further evaluated through an in vivo trial involving rats. In addition, the screening of metagenomic datasets of clinical population and rat models of depression and anxiety revealed an intriguing association/correlation between *B. adoles*centis load and these mental disorders.

## Results and discussion

### Distribution of GABA genes among the *Bifidobacterium* genus

**Figure 1:**
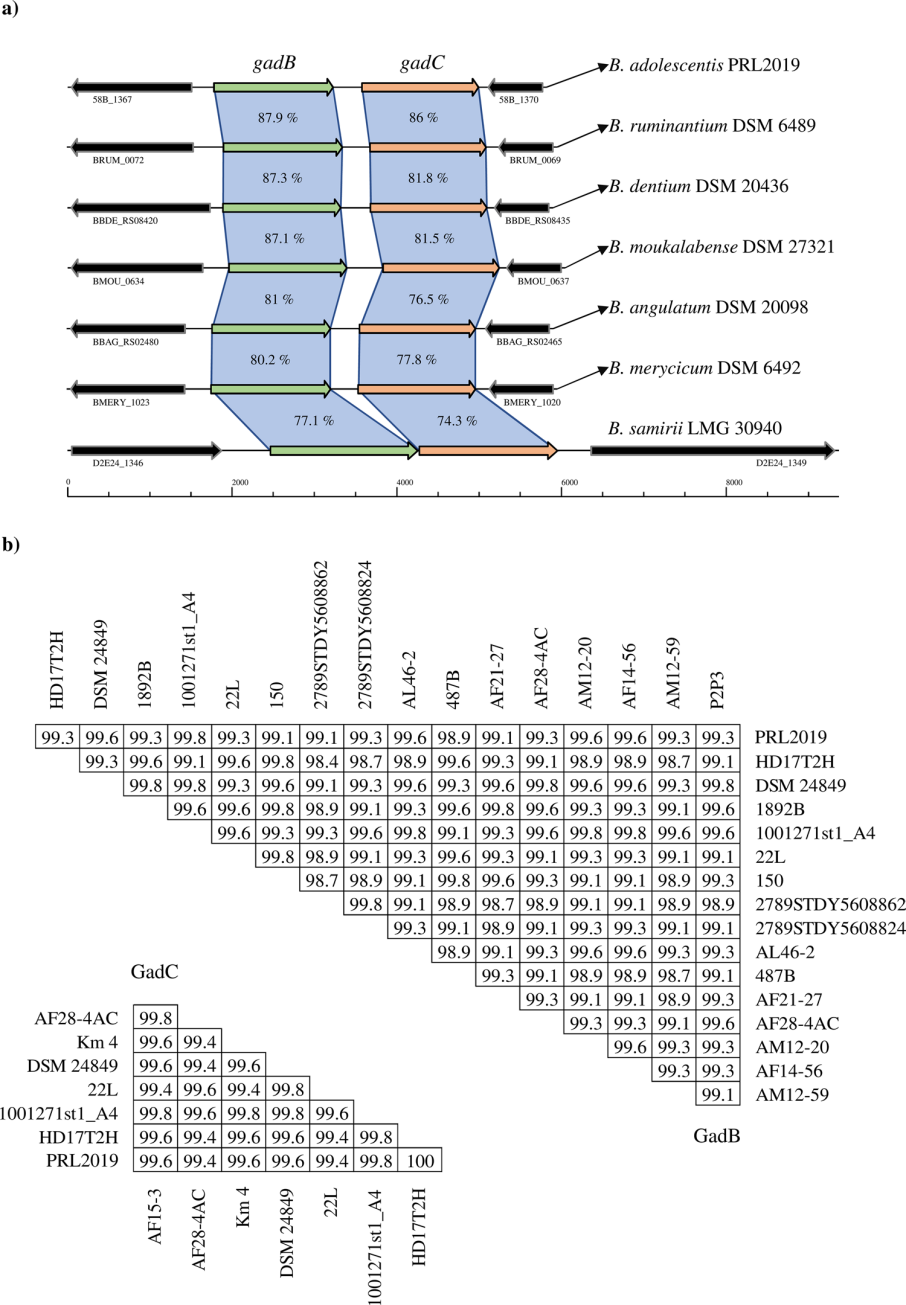
*Bifidobacterium* genetic map of GAD/GABA antiporter locus. Panel (**a**) displays genetic maps belonging to different *Bifidobacterium* species in which the locus has been identified. The *gad*B and *gad*C genes are highlighted with the relative color. Each arrow indicates an open reading frames (ORF), whereas the length of the arrow is proportional to the length of the predicted ORF. Panel (**b**) depicts the amino acid sequence identity values of GadB and GadC between the analyzed *B. adolescentis* genomes. Duplicates of both genes were removed to highlight non-redundant values between strains.

The ability to produce GABA by few gut-derived bifidobacterial taxa have been previously described^13^. Thus, a comprehensive screening of GABA production by bifidobacteria for each of the currently recognized species belonging to the genus *Bifidobacterium* was warranted. In order to fulfill this gap of knowledge a genetic survey involving 1,022 genomes from 81 (sub)species of the genus *Bifidobacterium*^14–16^, including taxa isolated from the gut of humans and animals, was performed to shed light into which taxa possess the appropriate genetic makeup for the synthesis of GABA. The dissected proteome of 1,022 bifidobacterial strains retrieved from the genomic NCBI database as well as our bifidobacterial genome database (Table S1), revealed that 81 strains encode for both GadB and GadC, encompassing seven different species, i.e., *B. adolescentis*, *Bifidobacterium angulatum*, *B. dentium*, *Bifidobacterium merycicum*, *Bifidobacterium moukalabense*, *Bifidobacterium ruminantium* and *Bifidobacterium samirii* (Table S3). Interestingly, four of the identified species that share the GAD/GABA antiporter locus belongs to members of the *B. adolescentis* phylogenetic group^14^, including 75 out of 81 analyzed genomes. Based on the sequence identity values obtained between the identified protein sequences, we observed a higher conservation among members of the *B. adolescentis* phylogenetic group, while lower values of identity were found in *B. angulatum*, *B. merycicum* and *B. samirii* taxa, which reflect their belonging to other bifidobacterial phylogenetic groups^14,15^ (Fig. 1a). Overall, among the identified bifidobacterial species sharing the GAD/GABA antiporter locus, *B. adolescentis*, *B. angulatum* and *B. dentium* are of human origins, while the other five taxa are usually associated with the gut of other mammals, such as monkeys and bovines^17–19^. Between the above listed taxa of human origins, members of the *B. adolescentis* species are the most scrutinized for both genomic and proved production of GABA^20–22^. Intriguingly, the high level of prevalence of GAD/GABA antiporter locus within the 50 *B. adolescentis* genomes analyzed (94%) (Table S3), coupled with the fact that such bifidobacterial species are occurring in the human gut^20,23^, suggests that this bifidobacterial taxon might represent a model GABA producer.

### Gut microbiota composition in depression and anxiety

**Figure 2:**
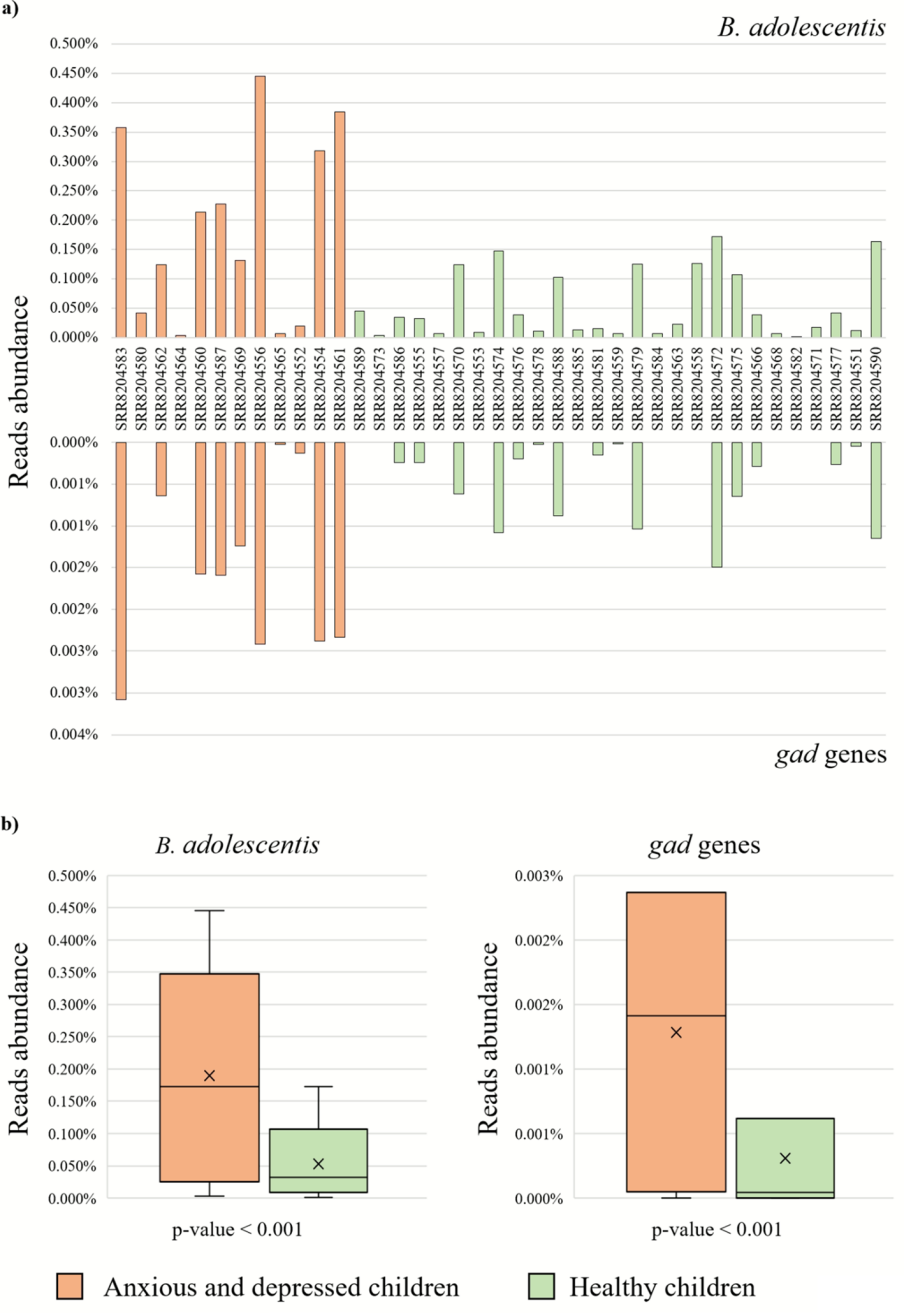
Relative abundance of *B. adolescentis* and *gad* genes within analyzed children gut microbiomes. Panel (**a**) shows the overall abundance of *B. adolescentis*- and *gad* genes-associated reads within the filtered children gut microbiome samples (PRJNA496479). The y-axis represents the percentage of reads identified, whereas the x-axis reports the sample numbers. Values associated to *gad* genes are reported in reverse order. The anxious and depressed children samples are represented as orange-colored bars, whereas healthy subjects in green. Panel (**b**) exhibits two Whisker plots based on relative abundances of *B. adolescentis* and *gad* genes in the gut microbiota data, which results in both chases with a *p* value of < 0.001 between depressed and healthy children (Student’s *t* test). The y axis shows the percentage of reads identified. Boxes represent 50% of the data set, distributed between the 1st and 3rd quartiles. The median divides the boxes into the interquartile range, while the X represents the mean. The lines extending vertically outside the boxes show the outlier range.

Since GABA, which is the primary inhibitory neurotransmitter known to counterbalance the action of the excitatory neurotransmitter glutamate, plays an important role in the treatment of anxiety and depressive disorders^24,25^, we decided to investigate the presence of *B. adolescentis* genomes and associated *gad* gene sequences in two public human gut microbiome datasets related to these illnesses (PRJNA496479 and PRJNA474710). Thus, metagenomic samples collected from children (PRJNA496479) were screened for reads corresponding to *gad* genes and *B. adolescentis* chromosome sequences, unveiling dissimilar profiles between samples (Fig. 2). The number of metagenomic reads belonging to *B. adolescentis* ranged from 76,127 to none, with higher values especially in samples belonging to anxious and depressed children (*t* test *p* value < 0.001, df = 37, Cohen's d = 0.97 and effect-size r = 0.43) (sample size estimation of 12 between groups, based on *B. adolescentis* abundance) (Fig. 2). Accordingly, metagenomic reads belonging to *gad* genes were found to be statistically higher in the samples displaying higher abundance of *B. adolescentis* (*t* test *p* value < 0.001, df = 37, Cohen's d = 1.02 and effect-size r = 0.45) (Fig. 2). Therefore, these data highlighted a clear correlation between the higher relative abundance of *B. adolescentis* sequences, together with related *gad* genes, and children with subclinical symptoms of anxiety and depression. In contrast, metagenomic samples from rats (PRJNA474710) displayed the complete absence of any trace of sequences related to *B. adolescentis* chromosome and *gad* genes. Such finding could be explained by the fact that *B. adolescentis* are not naturally occurring in the ceca of rats^23^. Based on these results, *B. adolescentis* was found to be an excellent model organism to investigate its ability to produce GABA in the gut environment.

### Production of GABA by *B. adolescentis* strains

**Figure 3:**
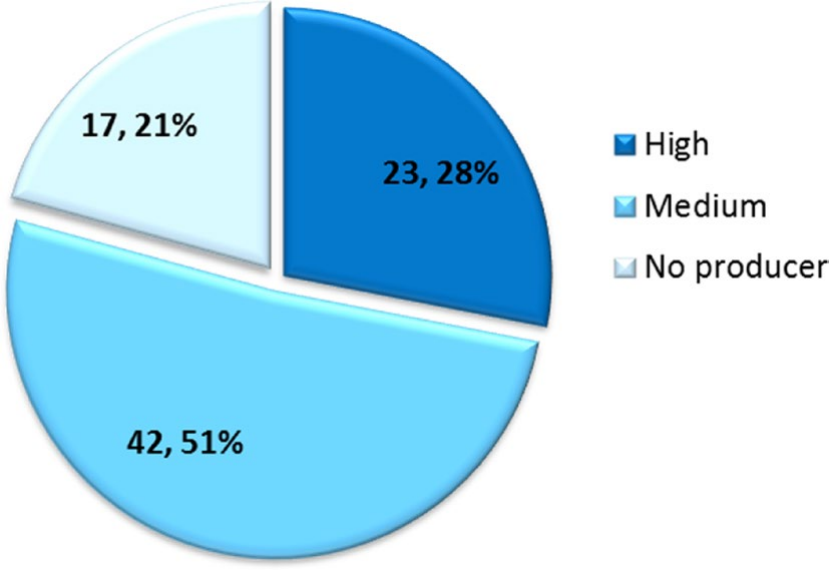
*B. adolescentis* distribution according to the production of GABA quantified by means of HPLC.

In order to investigate the production of GABA in *B. adolescentis* species, a collection of 82 bifidobacterial strains was scrutinized for this feature employing an in vitro approach*.* The investigated strains were mainly isolated from fecal samples or colon biopsy of healthy humans (Table 1). In accordance to the in silico data previously described, in vitro GABA production was revealed as a frequent trait of *B. adolescentis* taxon, since 79% of the tested *B. adolescentis* strains displayed the ability to transform the precursor monosodium glutamate (GMS) to GABA. Specifically, 23% of all the tested *B. adolescentis* strains were classified as high GABA producers, as they were capable to efficiently convert more than 65% of the precursor to GABA (Fig. 3). In view of these results, two representative strains classified as high GABA producers, i.e. *B. adolescentis* PRL2019 and *B. adolescentis* HD17T2H, were chosen as model bifidobacterial strains to further investigate this intriguing metabolic feature in an in vivo model.

**Table 1:** GABA production levels determined in overnight cultures from the 82 *Bifidobacterium* strains included in this work.

Species	Strain	Strain origin	[GABA] mM		% GMS conversion to GABA	
			Average	SD	Average	SD
*B. adolescentis*	14B	Intestine of adult	8.77	0.434	80.755	3.999
*B. adolescentis*	153B	Intestine of adult	1.72	0.534	15.877	4.921
*B. adolescentis*	1BCM1	Colon biopsy	6.04	2.272	55.587	20.918
*B. adolescentis*	1CCM5	Colon content	5.38	2.126	49.489	19.573
*B. adolescentis*	22L	Human milk	1.92	0.367	17.707	3.375
*B. adolescentis*	235B	Intestine of adult	6.78	0.726	62.397	6.687
*B. adolescentis*	236B	Intestine of adult	9.16	1.914	84.355	17.622
*B. adolescentis*	2BCM1	Colon biopsy	3.73	1.858	34.358	17.106
*B. adolescentis*	2BCM2	Colon biopsy	7.37	0.816	67.855	7.511
*B. adolescentis*	2CCM6	Colon content	5.67	0.862	52.196	7.940
*B. adolescentis*	2CCM7	Colon content	5.62	1.064	51.787	9.798
*B. adolescentis*	42B	Human faeces	4.60	0.328	42.314	3.023
*B. adolescentis*	487B	Human faeces	4.45	2.076	40.928	19.111
*B. adolescentis*	4CCM2	Colon content	2.82	0.887	25.944	8.170
*B. adolescentis*	50B	Intestine of adult	4.30	0.628	39.619	5.780
*B. adolescentis*	53B	Intestine of adult	8.62	0.614	79.410	5.656
*B. adolescentis*	55B	Intestine of adult	5.15	0.493	47.407	4.536
*B. adolescentis*	56B	Intestine of adult	5.19	0.193	47.798	1.774
*B. adolescentis*	57B	Intestine of adult	5.98	0.537	55.014	4.944
*B. adolescentis*	PRL2019	Intestine of adult	7.06	0.213	64.965	1.963
*B. adolescentis*	61B	Intestine of adult	4.50	0.254	41.447	2.342
*B. adolescentis*	62B	Intestine of adult	4.26	0.286	39.204	2.636
*B. adolescentis*	6BCM1	Colon biopsy	7.80	0.366	43.102	39.418
*B. adolescentis*	6CCM3	Colon content	7.13	0.803	65.673	7.390
*B. adolescentis*	703B	Human faeces	0.59	0.008	5.454	0.070
*B. adolescentis*	70B	Human faeces	3.59	0.115	33.065	1.059
*B. adolescentis*	712B	Human faeces	0.91	0.232	8.369	2.135
*B. adolescentis*	713B	Intestine of adult	4.53	0.676	41.696	6.224
*B. adolescentis*	714B	Intestine of adult	0.86	0.079	7.881	0.731
*B. adolescentis*	740B	Intestine of adult	0.71	0.059	6.496	0.545
*B. adolescentis*	74B	Intestine of adult	8.31	0.939	76.539	8.649
*B. adolescentis*	75B	Intestine of adult	5.31	0.839	29.338	27.333
*B. adolescentis*	76B	Intestine of adult	6.31	1.565	58.064	14.410
*B. adolescentis*	77B	Intestine of adult	3.33	0.356	30.679	3.280
*B. adolescentis*	780B	Intestine of adult	0.73	0.012	6.712	0.108
*B. adolescentis*	796B	Intestine of adult	4.35	0.419	40.034	3.857
*B. adolescentis*	79B	Intestine of adult	2.05	0.088	12.611	10.937
*B. adolescentis*	809B	Intestine of adult	7.73	0.542	71.159	4.990
*B. adolescentis*	856B	Intestine of adult	0.73	0.022	6.690	0.201
*B. adolescentis*	859B	Intestine of adult	0.64	0.017	5.875	0.161
*B. adolescentis*	951B	Intestine of adult	2.11	0.364	19.421	3.353
*B. adolescentis*	952B	Intestine of adult	1.11	0.385	10.219	3.549
*B. adolescentis*	954B	Intestine of adult	3.02	0.333	27.779	3.063
*B. adolescentis*	971B	Intestine of adult	1.82	0.095	16.745	0.871
*B. adolescentis*	AD2-8	Human faeces	5.71	1.839	52.601	16.936
*B. adolescentis*	AL12-4	Human faeces	0.64	0.072	5.889	0.659
*B. adolescentis*	HD17T1d	Human faeces	5.75	0.902	52.918	8.301
*B. adolescentis*	HD17T1h	Human faeces	0.87	0.036	8.024	0.332
*B. adolescentis*	HD17T2h	Human faeces	9.43	1.492	86.802	13.741
*B. adolescentis*	HD17T3h	Human faeces	0.97	0.027	8.959	0.247
*B. adolescentis*	HD17T9h	Human faeces	6.54	0.506	60.201	4.655
*B. adolescentis*	HD19T1h	Human faeces	4.29	0.692	39.526	6.367
*B. adolescentis*	HD19T2d	Human faeces	8.47	1.033	77.998	9.507
*B. adolescentis*	HD19T3h	Human faeces	2.85	0.209	26.263	1.921
*B. adolescentis*	HD23T1h	Human faeces	8.01	1.371	73.779	12.621
*B. adolescentis*	HD23T3d	Human faeces	3.42	0.819	31.461	7.539
*B. adolescentis*	HD23T4d	Human faeces	3.87	0.202	35.595	1.862
*B. adolescentis*	HD23T4h	Human faeces	5.03	0.140	46.349	1.289
*B. adolescentis*	HD23T6h	Human faeces	6.18	1.348	67.461	3.516
*B. adolescentis*	HD23T8h	Human faeces	5.25	0.290	48.347	2.669
*B. adolescentis*	HD24T1h	Human faeces	3.81	0.168	35.061	1.549
*B. adolescentis*	HD24T5h	Human faeces	9.32	0.367	85.788	3.379
*B. adolescentis*	HD24T7h	Human faeces	8.44	0.233	77.694	2.142
*B. adolescentis*	HD28T1d	Human faeces	7.45	1.133	68.605	10.431
*B. adolescentis*	HD28T2d	Human faeces	0.81	0.077	7.481	0.710
*B. adolescentis*	HD28T7h	Human faeces	0.66	0.079	6.049	0.729
*B. adolescentis*	HD35T1h	Human faeces	5.24	0.156	48.250	1.439
*B. adolescentis*	HD35T1h	Human faeces	7.96	1.541	85.557	0.072
*B. adolescentis*	HD35T2d	Human faeces	5.66	0.677	52.066	6.237
*B. adolescentis*	HD35T4d	Human faeces	5.82	0.708	53.553	6.517
*B. adolescentis*	HD35T5h	Human faeces	6.49	1.448	59.745	13.331
*B. adolescentis*	HD36T1h	Human faeces	0.87	0.066	8.052	0.605
*B. adolescentis*	HD36T2d	Human faeces	1.14	0.001	10.528	0.007
*B. adolescentis*	HD36T4h	Human faeces	0.94	0.082	8.609	0.755
*B. adolescentis*	HD36T6h	Human faeces	1.02	0.059	9.391	0.547
*B. adolescentis*	HD36T8h	Human faeces	0.91	0.008	8.369	0.078
*B. adolescentis*	HD4T2h	Human faeces	8.73	0.953	80.332	8.774
*B. adolescentis*	LMG10502	Culture collection, adult intestine	0.66	0.044	6.031	0.401
*B. adolescentis*	LMG10733	Culture collection, adult intestine	0.66	0.018	4.034	3.495
*B. adolescentis*	LMG10734	Culture collection, adult intestine	2.82	0.864	25.942	7.959
*B. adolescentis*	LMG11579	Culture collection, bovine rumen	1.35	0.275	12.403	2.529
*B. adolescentis*	LMG18897	Culture collection, human feces	5.94	0.171	54.670	1.574
*B. moukalabense*	DSM27231	Faeces of a wild lowland gorilla (*Gorilla gorilla*)	7.41	0.272	70.058	3.174
*B. stercoris*	JCM15918	Culture collection, human faeces	1.62	0.100	14.9381	0.9226
*B. angulatum*	LMG11039	Culture collection, human feaces	2.78	0.297	25.5759	2.7344
*B. dentium*	LMG11045	Human dental caries	5.57	0.056	51.327	0.517
*B. merycicum*	LMG11341	Culture collection, bovine rumen	0.62	0.014	5.747	0.133
*B. ruminatium*	LMG21811	Culture collection, bovine rumen	0.64	0.017	5.902	0.156

### Genetic features of *B. adolescentis* PRL2019 and *B. adolescentis* HD17T2H

The genome sequence length of selected representative strains classified as high GABA producers, namely *B. adolescentis* PRL2019 and *B. adolescentis* HD17T2H, consist of 2,212,477 and 2,163,875 bp with an average G + C content of 59.17% and 59.23%, respectively, which are similar to those of other sequenced bifidobacterial genomes, being consistent with the range of G + C mol % values previously described for Actinobacteria^26^ (Table 2). Furthermore, the genome of PRL2019 and HD17T2H possess 54 and 55 tRNA genes, respectively, and both genomes encompass four rRNA gene operons. Identification of protein-coding sequences revealed 1,796 open reading frames (ORFs) in PRL2019 strain and 1,753 ORFs in HD17T2H strain. Chromosome sequences of both strains were scrutinized allowing identifying genes encoding glutamate decarboxylase (*gad*B) and glutamate/GABA antiporter (*gad*C). The resulting amino acid sequences were compared to those of GadB and GadC belonging to 47 *B. adolescentis* strains possessing the GAD/GABA antiporter locus (Table S3). Sequence alignments highlighted GadB as a conserved protein among the *B. adolescentis* species, with sequence identities ranging from 98.4% to 100% (Fig. 1b). Moreover, GadC was identified as an even more highly conserved protein, sharing an amino acid identity sequence ranging from 99.4 to 100% between the analyzed *B. adolescentis* predicted proteomes (Fig. 1b). Additionally, based on search for homologous genes, we also identified in both genomes of *B. adolescentis* PRL2019 and HD17T2H the gene *pdxST* involved in vitamin B_6_ metabolism in bifidobacteria. In particular, pyridoxal 5′-phosphate (PLP), the metabolically active form of vitamin B_6_, represents an important cofactor in the biosynthesis of several neurotransmitters, including GABA^27,28^.

**Table 2:** General genetic features.

	*B. adolescentis* PRL2019	*B. adolescentis* HD17T2H
Biological origin	Human gut	Human feces
Average coverage	279	91
Number of assembled contigs	1	12
Genome length (pb)	2,212,477	2,163,875
Average GC percentage	59.17	59.23
Number of predicted ORFs	1,796	1,753
tRNA	54	55
rRNA	4	4*
Accession number	PRJNA628852	PRJNA628660

*Predicted number of rRNA loci.

### GABA production of *B. adolescentis* strains in a rat model

**Figure 4:**
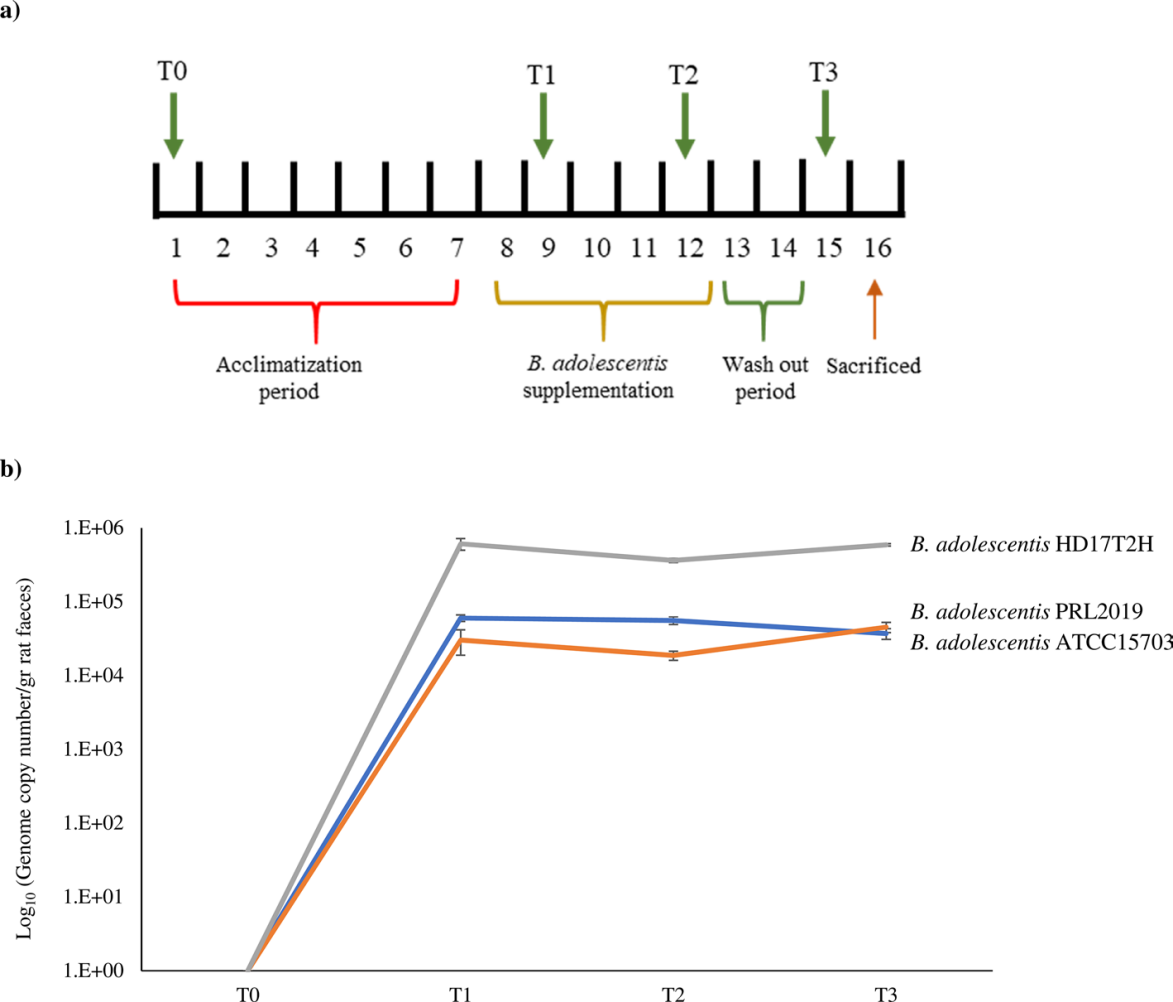
Schematic representation of in vivo trials. Panel (**a**) displays the schedule of the experimental procedures. Panel (**b**) shows the average of DNA presence of the *B. adolescentis* strains in faecal samples observed during the bifidobacterial administration. Each point represents the average of the log-population size ± standard deviation for eight rats.

**Figure 5:**
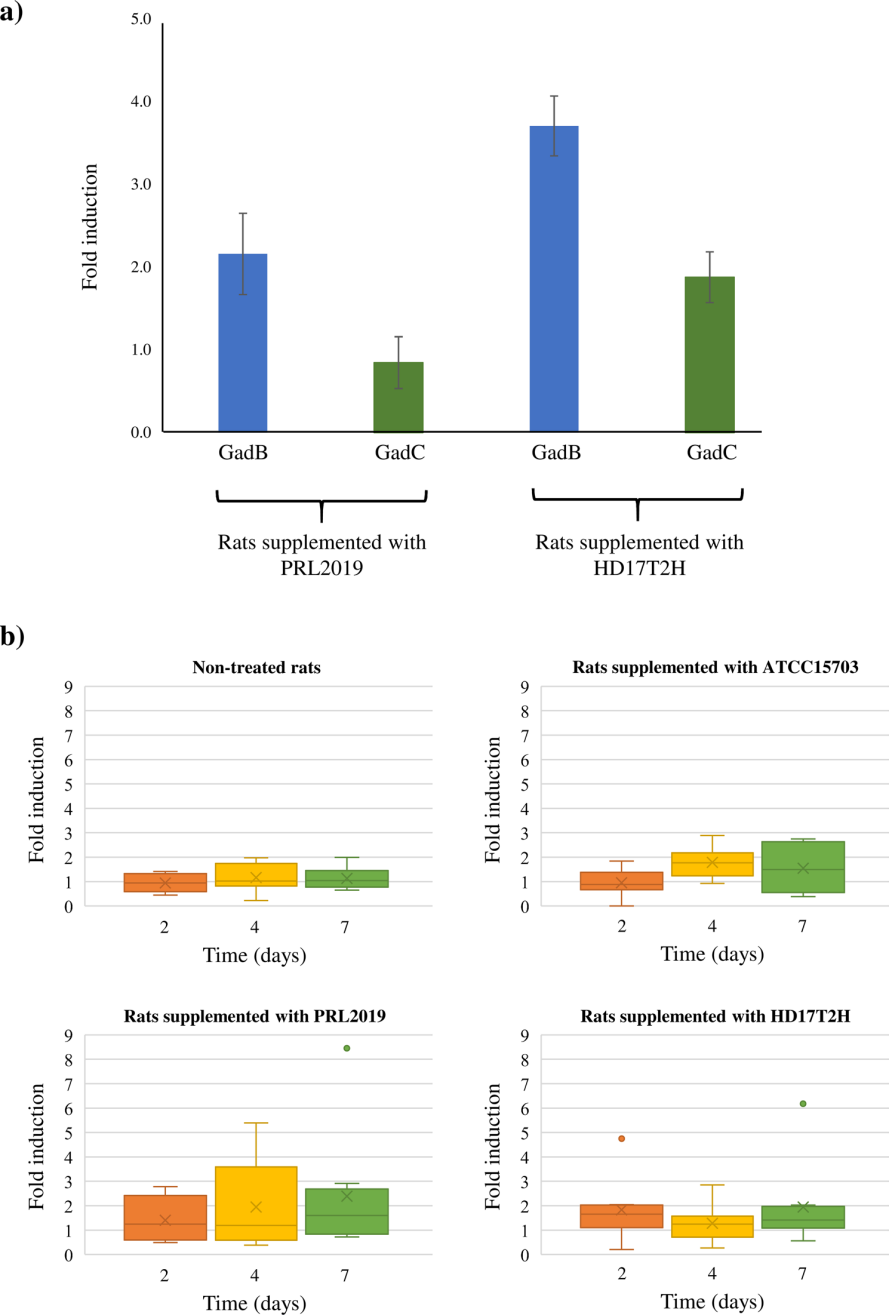
*Gad*B and g*ad*C gene expressions and GABA levels in rat feces. Panel (**a**) highlight the expression of *gad*B and *gad*C genes under in vivo conditions. Data are expressed as means ± standard deviation. Each experiment was performed in triplicate. The y axis represents the level of expression as normalized expression (ΔΔCt) in respect to the housekeeping *rpoB* and *atpB* genes. Panel (**b**) shows the fold induction of GABA in faeces of rats non-treated and treated for 5 days with *B. adolescentis* ATCC15703, *B. adolescentis* PRL2019 or *B. adolescentis* HD17T2H in respect to the GABA basal level in the corresponding T_0_. Box-plot represents the median (bold line), interquartile range (box), mean (X) and minimum and maximum values.

Three groups of rats (*Rattus norvegicus*) were supplemented for 5 days with a single daily dose of 10^9^ colony forming unit (CFU) of *B. adolescentis* strains isolated from the human gut, i.e. *B. adolescentis* ATCC15703, *B. adolescentis* PRL2019 and *B. adolescentis* HD17T2H (Fig. 4a). Notably, as above described, the genome of *B. adolescentis* ATCC15703 lacks *gad*B and *gad*C genes (Table S2). Furthermore, a fourth group of rats, representing the control group, was supplemented with a sucrose solution without any bifidobacterial strains. Subsequently, the abundance of *B. adolescentis* ATCC15703, *B. adolescentis* PRL2019 and *B. adolescentis* HD17T2H was monitored during the experiment using a qPCR approach based on strain-specific primers. Interestingly, data collected from the qPCR analysis revealed an estimated abundance of all supplemented *B. adolescentis* strains ranging from 10^4^ to 10^5^ CFU/gr (Fig. 4b). These data highlighted a stable bifidobacterial abundance between samples collected from T_1_ to T_3_ that correspond with the bacterial supplementation (see materials and methods) (Fig. 4b). Furthermore, in order to investigate the expression level of genes involved in the GABA metabolism of PRL2019 and HD17T2H, we performed transcription profiling of *gad*B and *gad*C genes using a qRT-PCR approach on rats’ feces collected at T_3_. Interestingly, the transcription level of PRL2019 and HD17T2H *gad* genes revealed that *gad*B expression was significantly enhanced, ranging from 1.5- to sevenfold induction, while the expression of *gad*C ranged from 0.1 to fourfold induction (Fig. 5a). The enhanced expression of genes belonging to the GAD/GABA antiporter locus, revealed that *B. adolescentis* PRL2019 and *B. adolescentis* HD17T2H are able to stimulate the GABA production in rat model.

In order to evaluate the GABA level in rats involved in these experiments, we performed an ELISA assay among fecal samples collected at different time points, i.e. T_0_, T_1_, T_2_ and T_3_. Interestingly, the concentration of GABA (μg/g) seemed to increase in rats treated with *B. adolescentis* PRL2019 and *B. adolescentis* HD17T2H, but no statistical differences were found with respect to rats treated with no-GABA producer strain *B. adolescentis* ATCC15703 and with respect to rats not supplemented by *B. adolescentis* strains (control group) (Fig. S1). The normalized concentration of GABA, normalized respect to the T_0_ data, revealed higher GABA levels in rats treated with GABA-producer *B. adolescentis* strains, but also in the non-producer ATCC15703 strain when compared with the control group (Fig. 5b). In particular, rats treated with *B. adolescentis* PRL2019 revealed a twofold increase of GABA level after 4 days of treatment, while rats treated with *B. adolescentis* HD17T2H highlighted an enhancement of 1.4-fold after the first 2 days of treatment. Despite the higher abundance of *B. adolescentis* HD17T2H (Fig. 4b) and the higher *gad* genes expression fold induction in respect to PRL2019 (Fig. 5a), the GABA concentration at T_3_ was lower (Fig. 5b), suggesting that the amount of in vivo produced GABA was not proportional between strains. Furthermore, the increased concentration of GABA even in rats fed with *B. adolescentis* ATCC15703 that does not harbor *gad* genes, could suggest that the administration of this species of *Bifidobacterium* could modulate the intestinal microbiota of rats favoring those endogenous populations able to synthesize this neurotransmitter.

## Conclusions

In the current study, we performed a comprehensive in silico survey of 1,022 bifidobacterial genomes highlighting the genetic arsenal requested for the synthesis of GABA in seven different bifidobacterial species, i.e. *B. adolescentis*, *B. angulatum*, *B. dentium*, *B. merycicum*, *B. moukalabense*, *B. ruminantium* and *B. samirii*. Intriguingly, *B. adolescentis* strains showed the highest level of prevalence of *gad* genes in their genomes, suggesting this bifidobacterial taxon as a model GABA producer within the *Bifidobacterium* genus. Furthermore, metagenomics-based analyses involving datasets collected from children with subclinical symptoms of depression and anxiety revealed an intriguing association/correlation with reads belonging to *B. adolescentis* as well as *B. adolescentis gad* genes.

The in vitro screening of 82 *B. adolescentis* strains isolated from the human gut allowed to highlight those exhibiting the highest performances in the synthesis of GABA. Among *B. adolescentis* isolates, strains PRL2019 and HD17T2H were employed in an in vivo trial, highlighting an enhanced expression of GABA level in rats following the treatment with these bacteria. However, in vivo trials with animal models of anxiety/depression disorders will need to be performed in order to further support these findings and validate the role of *B. adolescentis* in the modulation of gut–brain axis signaling. Nonetheless, the achieved results contribute the expanding of the current knowledge about a possible role of *B. adolescentis* in the modulation of the gut microbiota-brain axis, since PRL2019 and HD17T2H strains represent intriguing GABA-producing gut microbes isolated from humans.

## Materials and methods

### *Bifidobacterium adolescentis* strains and growth conditions

All strains used in this study were cultivated in an anaerobic atmosphere (10% H_2_, 10% CO_2_ and 80% N_2_) in an anaerobic MG500 chamber (Don Whitley Scientific, West Yorkshire, United Kingdom) on De Man-Rogosa-Sharp (MRS) broth (BD-Difco Biosciences, San Diego, CA) supplemented with 0.25% (w/v) l-cysteine hydrochloride (Sigma-Aldrich) and incubated at 37 °C for variable times (Table 1).

### Measurement of GABA production

To determine GABA production, strains were subcultured in MRS supplemented with 2 mM monosodium glutamate (GMS, Sigma-Aldrich) and grown for 48 h anaerobically at 37ºC. GABA production was evaluated by HPLC on cell-free supernatants following diethyl ethoxymethylenemalonate (DEEM, Sigma-Aldrich) derivatization according to the following indications^29^. After centrifugation (18,000* g* for 10 min), supernatants were filtered through a syringe filters (13 mm diameter, 0.22 µm pore size, PTFE membrane, VWR International, Radnor, PA, USA). Aliquots of 100 µl were thoroughly mixed by vortexing with 175 µl of borate buffer (1 M boric acid, pH 9.0), 75 µl methanol, 3 µl DEEM and 2 µl of 2-l-amino adipic acid (stock solution at 2 mgml^−1^) (Sigma-Aldrich), as an internal standard. Mixtures were held in an ultrasound water bath at 30º C for three 15 min cycles. Then samples were maintained at 70ºC in a water bath for 2 h to remove DEEM excess. Finally, samples were centrifuged for 5 min at 11,000* g* and supernatants were further filtered through 0.22 µm membranes.

GABA was determined by reverse-phase (RP)-HPLC in the Ascentis C18 (250 × 4.6 mm, 5 μm) column coupled with a pre-column Supelguard Ascentis C18 (20 × 4.0,0 mm) (Supelco, Sigma-Aldrich, St. Louis, MO), using a chromatographic system composed of the Alliance 2,695 separation module, the UV–visible PDA 2,996 detector and the acquisition/analysis software Empower (Waters, Milford, MA, USA). Separation was carried out at 35ºC with a gradient of the mobile phase: 25 mM acetate buffer pH 6.7 plus 0.02% sodium azide (eluent A), acetonitrile (eluent B) and methanol (eluent C)^30^. Samples (5 l) were injected, separated at 1 ml min^−1^ flow rate (total rum 100 min) and the GABA was detected at 280 nm. Quantification was performed using external calibration pattern using known concentrations of GABA standard (Sigma), submitted to the same derivatization procedure, to obtain the corresponding linear regression equation (R^2^ > 0.99). All determinations were performed, at least, in two independent biological replicates.

### Genome sequencing and assemblies

Based on the results achieved from the production of GABA between 82 *B. adolescentis* strains, two representative strains classified as high GABA producers namely *B. adolescentis* PRL2019 and *B. adolescentis* HD17T2H, were submitted to shotgun genome sequencing. DNA extracted from *B. adolescentis* PRL2019 and *B. adolescentis* HD17T2H cultures was subjected to whole-genome sequencing using MiSeq (Illumina, UK) at GenProbio srl (Parma, Italy) according to the supplier’s protocol (Illumina, UK). Moreover, in order to improve the genome quality of *B. adolescentis* PRL2019, its DNA was extracted and submitted to whole-genome sequencing using a MinION approach (Oxford Nanopore, UK) at GenProbio srl (Parma, Italy) according to the supplier’s protocol (Oxford Nanopore, UK). Fastq files of the paired-end reads obtained from targeted genome sequencing of isolated strains were utilized as input for genome assemblies through the MEGAnnotator pipeline^31^. SPAdes software was used for de novo assembly of each *Bifidobacterium adolescentis* genome sequence^32,33^, while open reading frames (ORFs) were predicted using Prodigal^34^. The coverage depth of these newly isolated *B. adolescentis* chromosomes ranged from 91- to 279-fold, which upon assembly generated 12 contigs and a complete chromosome sequence, respectively.

### GAD/GABA antiporter locus identification

We retrieved the proteome of 1,022 *Bifidobacterium* strains from the National Center for Biotechnology Information (NCBI) public database (Table S1). Accordingly, we assessed which bifidobacterial species encode the genes required for GABA production by means of local alignment search against the NCBI bifidobacterial reference glutamate decarboxylase (GadB) and glutamate/GABA antiporter (GadC) amino acid sequences (Accession: ADB10338.1 and VEG24324.1). Putative GadB and GadC proteins of the 1,022 *Bifidobacterium* strains were identified by means of BLASTP (cutoff E value, 1 × 10^−30^ and 50% identity over at least 80% of both protein sequences).

### Shotgun metagenomic screening of *B. adolescentis* and *gad* gene sequences

In order to investigate the presence of *B. adolescentis* and to explore the occurrence of *gad* genes into the microbiota of individuals exhibiting depression and anxiety behaviors, we analyzed two public metagenomic datasets related to these illnesses (PRJNA496479 and PRJNA474710). In this context, we collected the metagenomic data of a cohort of early school-aged children with a combination of subclinical mental health symptoms of depression and anxiety (PRJNA496479) and those of a well-characterized model of stress vulnerable Sprague Dawley rats showing depressive- and anxiety-like behaviors due to social defeat (PRJNA474710). Each data set was filtered to obtain only high quality reads (minimum mean quality score 20; window size 5; quality threshold 25; minimum length 80) using the fastq-mcf script (https://expressionanalysis.github.io/ea-utils/). The resulting reads were aligned against the *Homo sapiens* and *Rattus norvegicus* genomes using the Burrows-Wheeler Aligner program^35^ (BWA-MEM algorithm with trigger reseeding, 1.5; minimum seed length, 19; matching score, 1; mismatch penalty, 4; gap open penalty, 6; and gap extension penalty, 1) and further processed with the SAMtools software package^36^ in order to remove human and rats reads. Finally, the filtered reads were used to identify *B. adolescentis*-associated reads within the data set for each sample by means of Bowtie2^37^ through multiple-hit mapping and a “very sensitive” policy. The mapping was performed using a minimum score threshold function (–score-min C, -13,0) in order to limit reads of arbitrary length to two mismatches and retain those matches with at least 98% full-length identity. The software employed to calculate read counts corresponding to bifidobacterial genes was HTSeq^38^, running in union mode.

### Experimental design of the in vivo trials

Experiments involved 5-month-old male wild-type Groningen rats (*R. norvegicus*). This rat strain, originally derived from the University of Groningen (The Netherlands), was bred in the animal facility of the University of Parma under standard conditions. From the initiation of the experiments, rats were housed individually in polymethyl methacrylate (Plexiglas) cages (39 cm × 23 cm × 15 cm). Rats were kept in rooms with controlled temperature (22 ± 2 °C) and humidity (60 ± 10%) and maintained in a 12/12 light/dark cycle (light on from 19:00 to 7:00 h), with food and water ad libitum. The first week represented an acclimatization period, during which rats continued to consume a standard chow diet supplemented with an oral administration of 500 µl of sucrose solution (2%) in order to adapt to drink from a syringe. For the following 5 days, rats (n = 32) were randomized to 4 groups and orally supplemented using a syringe with: (1) *B. adolescentis* ATCC15703; (2) *B. adolescentis* PRL2019; (3) *B. adolescentis* HD17T2H; (4) sucrose solution only (i.e., negative control) (Table S2). The treatment with *B. adolescentis* strains was daily administered at 10^9^ CFU per rat by syringe. Before the treatment, microbial cultures were cultivated as previously described, and fecal samples of rats were analyzed to ensure the absence of *B. adolescentis* strains by means of specific primers. Subsequently, bacterial cultures were harvested by centrifugation (3,000 rpm for 8 min), washed and resuspended in 500 µL of 2% (w/v) sucrose solution. The viable count of each inoculum was determined by retrospective plating on MRS. In order to evaluate bifidobacterial colonization fecal samples were collected at four different time points. The first sample collection was performed before the oral administration of bifidobacteria (T_0_), in order to access the baseline concentration of GABA in each rat. Then, we collected fecal samples at 2, 4 and 7 days (T_1_, T_2_ and T_3_) to cover with multiple sampling the days the oral bifidobacterial supplementation (Fig. 4a). Faeces were stored at − 80 °C until use.

### DNA extraction and qPCR

Bacterial DNA extraction from rat’s fecal samples was performed following the manufacturer’s protocol of the QIAamp Fast DNA stool Mini Kit (Qiagen Ltd, Strasse, Germany). Bifidobacterial DNA presence was evaluated in rat’s fecal samples. Quantitative PCR (qPCR) was performed as described previously^39^. Strain specific primers were designed for the identification of different *B. adolescentis* strains in fecal samples. Primers Bado_PRL2019_fw (5′-GAGCAGGCAAGGACACTTTC-3′) and Bado_PRL2019_rev (5′-CTGAAGAGGCAAGCTTGAGG-3′) were used for *B. adolescentis* PRL2019; primers Bado_HD17T2M_fw (5′-CGGCTACAGGTTCGCTTATC-3′) and Bado_ HD17T2H_rev (5′-TTCCGCAGTAATTCGAGCTT-3′) were used for *B. adolescentis* HD17T2H; and Bado_ATCC15703_fw (5′-GGTGATTACGCAGCATCCTT-3′) and Bado_ATCC15703_rev (5′-CTTCCTCACAAACGTCAGCA-3′) were used for *B. adolescentis* ATCC15703. PCR products were detected with SYBR green fluorescent dye and amplified according to the following protocol: one cycle of 95 °C for 2 min, followed by 42 cycles of 95 °C for 15 s and Tm 62 °C for *B. adolescentis* PRL2019, 64 °C *B. adolescentis* HD17T2H and 60 °C *B. adolescentis* ATCC15703, for 30 s. The melting curve was 65 °C to 95 °C with increments of 0.5 °C/s. In each run, a negative control (no DNA) for each primer set was included.

### RNA extraction and qRT-PCR

In order to evaluate the expression of genes involved in GABA production, we have extracted the total RNA from faecal samples of rats. 0.4 g of stool sample were mixed to 1 mL of QIAzoL Lysis Reagent (Qiagen, UK) and were transferred in a sterile tube containing glass beads (Merck, Germany). The cells were lysed using Precellys 24 homogenizer (Bertin instruments, France). The protocol provides 2 min of stirring the mix alternating with 2 min of static cooling; this step was repeated three times. The cells were centrifuged at 12,000 rpm for 15 min and the upper phase was recovered. The RNA samples were purified using the RNAesy Mini Kit (Qiagen, UK) following the manufacturer’s protocol. RNA concentration and purity were evaluated by a Picodrop microliter spectrophotometer (Picodrop, UK). cDNA was synthesized and purified using the iScript cDNAsynthesis kit (Bio-Rad, CA, USA) according to the supplier’s instructions. Primers used for the normalization of the data were designed on housekeeping genes, i.e. *rpoB* and *atpB*, as described previously^40^, while for *gad*B gene were used primers GadB_fw (5′-CACATGCTCGCCGATCTATG-3′) and GadB_rev (5′-TCGACCGGCTCATACATACC-3′), whereas for *gad*C gene were used primers GadC_fw (5′-GTCTCGCTTCCATTCTGCTG-3′) and GadC_rev (5′-CGAACACATACGACAGGCTG-3′). qRT-PCR was performed using the CFX96 system (Bio-Rad, CA, USA). PCR products were detected with SYBR green fluorescent dye and amplified according to the following protocol: one cycle of 95 °C for 2 min, followed by 42 cycles of 95 °C for 15 s and 60 °C for 30 s. The melting curve was 65 °C to 95 °C with increments of 0.5 °C/s. In each run, a negative control (no cDNA) for each primer set was included. The expression ratio of the selected genes was calculated and analyzed using CFX Manager Expression software (Bio-Rad, CA, USA).

### GABA measurement in rat faeces

Faeces of each rat at different time points were diluted 1:10 (w/v) in milli-Q water in order to obtain faecal waters. Each sample was mixing until disaggregation of faeces and centrifuging at 5,000 rpm for 5 min and keeping the supernatant fraction. This aqueous fraction was used for quantification of GABA using the GABA ELISA kit (LDN Diagnostics, Germany) following manufacturer instructions. Dilution factor was taken into account for GABA calculation.

### Statistical analyses

SPSS software v. 25 (IBM, Italy) was used to perform statistical analysis between shogun metagenomic data of anxious and depressed children, and healthy subjects (BioProjects PRJNA496479) by Student’s *t* test. The sample size between groups was evaluated by means of Statulator (https://statulator.com/SampleSize/ss2M.html).

### Ethical statement

All experimental procedures and protocols involving animals were approved by the Italian Ministry of Health and the Veterinarian Animal Care and Use Committee of Parma University (Authorization Number 370/2018) and conducted in accordance with the European Community Council Directives dated 22 September 2010 (2010/63/UE).

## Supplementary information

Supplementary Information

Supplementary Tables

## Data Availability

Newly isolated *B. adolescentis* genomes were sequenced and deposited at DDBJ/ENA/GenBank under the accession numbers reported in Table 2 (BioProject No. PRJNA628660 and PRJNA628852).
