# Supplementary material for: Bifidobacterium adolescentis as a key member of the human gut microbiota in the production of GABA
Source: Sci Rep. 2020 Aug 24;10:14112. doi: 10.1038/s41598-020-70986-z (PMC7445748; doi:10.1038/s41598-020-70986-z)
Supplement: Supplementary file 1 — Supplementary Information [file 41598_2020_70986_MOESM1_ESM.docx]

***Bifidobacterium adolescentis* as a key member of the human gut microbiota in the production of GABA.**

Running title: *Bifidobacterium adolescentis* and GABA

Keywords: genomics, metagenomics, microbiota, bifidobacteria, gut-brain axis

Sabrina Duranti^1#^, Lorena Ruiz^2,3#^, Gabriele Andrea Lugli^1#^, Héctor Tames^2,3^, Christian Milani^1,4^, Leonardo Mancabelli^1^, Walter Mancino^1^, Giulia Longhi^5^, Luca Carnevali^6^, Andrea Sgoifo^4,6^, Abelardo Margolles^2,3^, Marco Ventura^1,4^, Patricia Ruas Madiedo^2,3^, Francesca Turroni^1,4^

#These authors contributed equally

Laboratory of Probiogenomics, Department of Chemistry, Life Sciences, and Environmental Sustainability, University of Parma, Parma, Italy^1^; Department of Microbiology and Biochemistry of Dairy Products, Instituto de Productos Lácteos de Asturias (IPLA)-Consejo Superior de Investigaciones Científicas (CSIC), Villaviciosa, Spain^2^; Instituto de Investigación Sanitaria del Principado de Asturias (ISPA), Oviedo, Asturias, Spain^3^; Microbiome Research Hub, University of Parma, Parma, Italy^4^; GenProbio srl Parma, Italy^5^; Stress Physiology Laboratory, Department of Chemistry, Life Sciences and Environmental Sustainability, University of Parma, Parma, Italy^6^

Correspondence:

Mailing address for Francesca Turroni, Laboratory of Probiogenomics, Department of Chemistry, Life Sciences, and Environmental Sustainability, University of Parma, Parco Area delle Scienze 11a, 43124 Parma, Italy. Phone: ++39-521-905644. Fax: ++39-521-905604. E-mail: francesca.turroni@unipr.it

Mailing address for Patricia Ruas Madiedo Microhealth Group, Department of Microbiology and Biochemistry of Dairy Products, Instituto de Productos Lácteos de Asturias – Consejo Superior de Investigaciones Científicas (IPLA-CSIC), Villaviciosa, Asturias, Spain, [ruas-madiedo@ipla.csic.es](mailto:ruas-madiedo@ipla.csic.es)

**
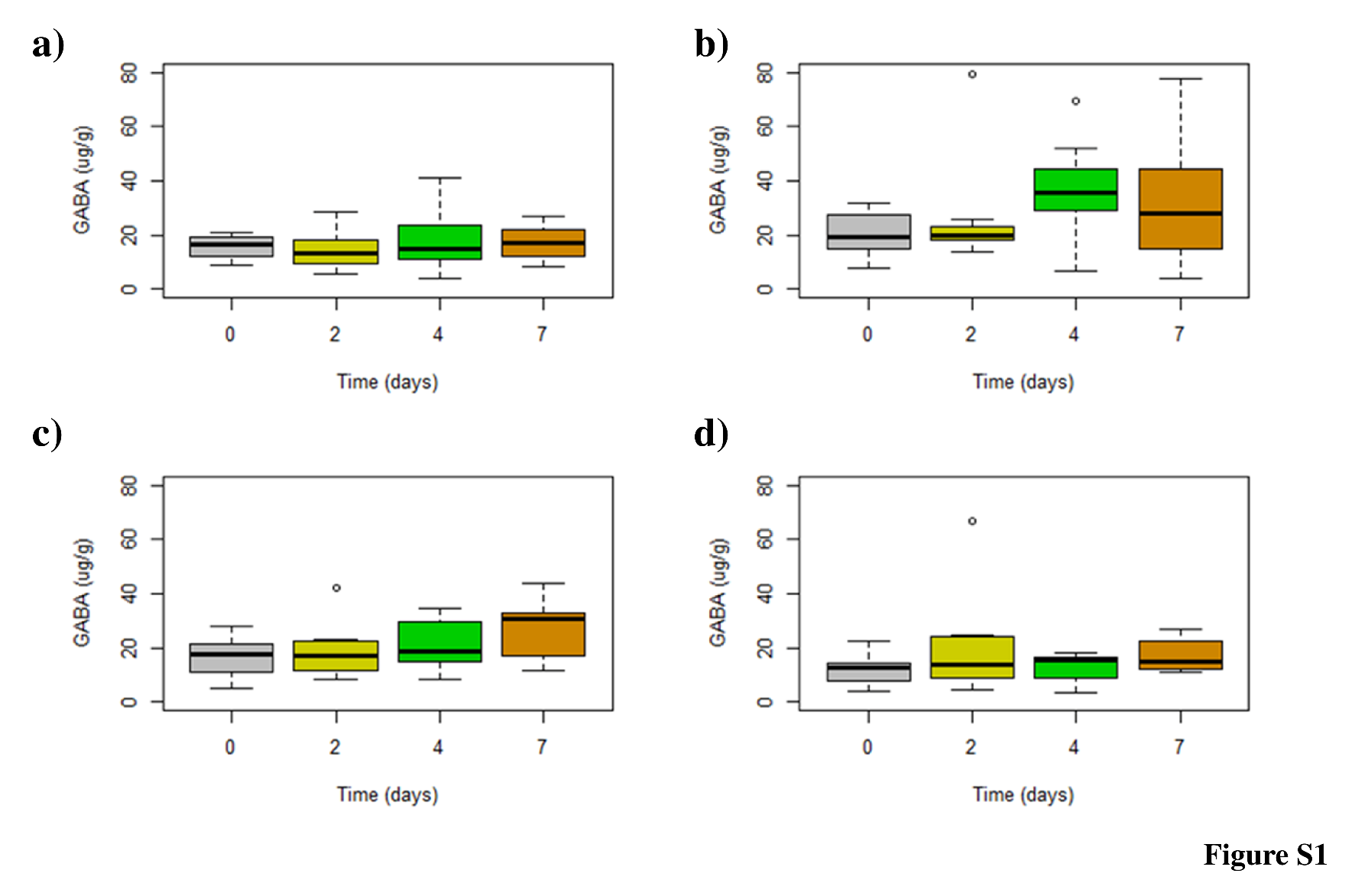
**

Figure S1. Concentration (μg/g) of GABA in faeces of rats non-treated (panel a) and treated with *B. adolescentis* ATCC15703 (panel b), *B. adolescentis* PRL2019 (panel c) and *B. adolescentis* HD17T2H (panel d). Box-plot represents the median (bold line), interquartile range (box) and minimum and maximum values.
